# Proton therapy and oral mucositis in oral & oropharyngeal cancers: outcomes, dosimetric and NTCP benefit

**DOI:** 10.1186/s13014-023-02317-1

**Published:** 2023-07-19

**Authors:** Sapna Nangia, Utpal Gaikwad, M. P. Noufal, Mayur Sawant, Manoj Wakde, Ashwathy Mathew, Srinivas Chilukuri, Dayananda Sharma, Rakesh Jalali

**Affiliations:** 1 Department of Radiation Oncology, Apollo Proton Cancer Centre, Dr. Vikram Sarabhai Instronic Estate, Taramani, Chennai, Tamil Nadu India; 2 Department of Medical Physics, Apollo Proton Cancer Centre, Chennai, Tamil Nadu India; 3 Department of Radiation Oncology, Tata Memorial Centre, Mumbai, India

**Keywords:** Proton, NTCP, Mucositis, Oral cavity, Oropharyngeal

## Abstract

**Introduction:**

Radiation-induced oral mucositis (RIOM), is a common, debilitating, acute side effect of radiotherapy for oral cavity (OC) and oropharyngeal (OPx) cancers; technical innovations for reducing it are seldom discussed. Intensity-modulated-proton-therapy (IMPT) has been reported extensively for treating OPx cancers, and less frequently for OC cancers. We aim to quantify the reduction in the likelihood of RIOM in treating these 2 subsites with IMPT compared to Helical Tomotherapy.

**Material and methods:**

We report acute toxicities and early outcomes of 22 consecutive patients with OC and OPx cancers treated with IMPT, and compare the dosimetry and normal tissue complication probability (NTCP) of ≥ grade 3 mucositis for IMPT and HT.

**Results:**

Twenty two patients, 77% males, 41% elderly and 73% OC subsite, were reviewed. With comparable target coverage, IMPT significantly reduced the mean dose and D32, D39, D45, and D50, for both the oral mucosa (OM) and spared oral mucosa (sOM). With IMPT, there was a 7% absolute and 16.5% relative reduction in NTCP for grade 3 mucositis for OM, compared to HT. IMPT further reduced NTCP for sOM, and the benefit was maintained in OC, OPx subsites and elderly subgroup.

Acute toxicities, grade III dermatitis and mucositis, were noted in 50% and 45.5% patients, respectively, while 22.7% patients had grade 3 dysphagia. Compared with published data, the hospital admission rate, median weight loss, feeding tube insertion, unplanned treatment gaps were lower with IMPT. At a median follow-up of 15 months, 81.8% were alive; 72.7%, alive without disease and 9%, alive with disease.

**Conclusion:**

The dosimetric benefit of IMPT translates into NTCP reduction for grade 3 mucositis compared to Helical Tomotherapy for OPx and OC cancers and encourages the use of IMPT in their management.

**Supplementary Information:**

The online version contains supplementary material available at 10.1186/s13014-023-02317-1.

## Introduction

Radiation-induced oral mucositis (RIOM), is a troubling acute side effect of radiotherapy for head-neck cancers (HNC), especially oral cavity (OC) and oropharyngeal (OPx) cancers. It occurs due to cellular depletion, loss of integrity of the mucosal barrier, inflammation, immune reaction and release of cytokines [1]. Apart from patient-related factors and concurrent systemic therapies, the radiation dose received by the oral mucosa (OM) is the most important factor in developing RIOM [2].

RIOM results in the requirement of analgesics including NSAIDs, opioids and gabapentinoids [3, 4]. In addition, it results in decreased oral intake, weight loss, interruption or modification of treatment, and the risk of feeding-tube insertion. The risks of infection, antibiotic usage and hospitalization are also higher in patients with oral mucositis, as is the risk of aspiration pneumonia, hypothesized as the causative factor of toxic deaths due to the mucositis dysphagia aspiration sepsis (MDAS) syndrome [5]. The impact of RIOM is felt on many QOL domains, including performance status, body-weight, pain, activity, recreation, swallowing, saliva, mood and anxiety [6].

While the potential of intensity-modulated-radiotherapy (IMRT) to reduce mucositis was recognized early in 2001, dose constraints were first defined only in 2008 by Narayan et al. [7]. The validity of these were confirmed in a randomized setting [8] and additional dose constraints have been identified as significant [9, 10]. Ascertaining a dose-volume-relationship has however been constrained by non-uniformity in delineation of the OM; the definition of OM proposed by Dean et al. limits this variability [11].

Modern radiotherapy techniques viz., IMRT, VMAT and Helical Tomotherapy (HT) aim to reduce radiation dose received by organs at risk (OARs) to limit normal tissue complication probability (NTCP), and improve the therapeutic ratio [12, 13]. Proton therapy (PT), with its particular physical characteristics, allows better sparing of OARs [14]. The conformality and convenience afforded by the pencil-beam-scanning (PBS) technique have led to an increase in the application of PT in HNC, with benefits being noted in various patient groups. Langendijk et al. have reported NTCP-based plan comparisons to identify patients who will benefit from PT [15]. The reduction in NTCP of oral mucositis and other side effects has been studied by Tambas et al. in a dosimetric analysis; the authors noted a 5–10% benefit with PT in all HNC subsites, the highest in hypopharyngeal—laryngeal tumours [16].

Our study aims to quantify the reduction in pertinent oral mucosal dose parameters, as well as NTCP for grade 3 RIOM, in OC and OPx cancer patients treated with PT compared with IMRT plans. We have included parotid tumours, treated with adjuvant PT as OC lesions as their radiation treatment resembles unilateral OC irradiation and has previously been studied with oral cavity cancers [17]. In addition, we report early toxicity and outcome data.

## Material and methods

We report the results of 22 consecutive patients with OC and OPx cancers treated with definitive or adjuvant (post-op) IMPT, from January 2019 to May 2020. Details of the planning process are documented in the Additional file 1. Primary and nodal clinical-target-volumes (CTVs) were delineated as previously reported [18, 19]. While there are separate guidelines for the delineation of the oral cavity [20] and oral mucosal surface [11], we opted for the latter, as proposed by Dean et al. [11] (Fig. 3, Additional file 1). The mucosa overlapping with the CTV was excluded to generate the spared oral mucosa (sOM). Dose constraints prescribed to this structure were similar to oral cavity constraints.

Rival IMRT (Helical Tomotherapy) and IMPT plans were generated on the Accuray Precision TPS, Version 2.0.1.1and Ray Search Raystation TPS, Version 9, respectively as detailed in the Additional file 1.

Proton therapy treatments were selected on the basis of dosimetric superiority of target coverage, or OAR sparing or both. Treatment was administered on a Proteus Plus machine (IBA, Louvain – La- Neuve, Belgium); the details are documented in the Additional file 1.

Acute adverse events were recorded according to the RTOG grading. The grade of mucositis within the treatment volume and outside, as assessed on clinical examination, was recorded separately.

Using the same plans, NTCP of ≥ grade 3 mucositis for IMPT and HT plans was also calculated using the following formula

NTCP Mucositis $={\left(1+\left(\frac{D50}{D}\right)k\right)}^{-1}$ , where D is the mean dose, D50 is 51GyE and k = 1. [9, 21].

Dosimetric data for all patients were collected and compiled using SPSS Software version 21, which was also used for statistical analysis. Dosimetric variables for OM and sOM for IMPT and HT plans were compared using paired t-test; two-tailed significance (p value) was calculated (Fig. 4; Additional file 1).

## Results

Patient demographics including the comorbidity index, frailty score and clinical details are summarized in Table 1. The median age of this population, comprising 22 patients, was 56 years (range 24 – 80 years);5 (23%) were female.

**Table 1:** Demographics and salient clinical features

Age (median)	56 Years (24–80) 9 (41%) were age > / = 65 years
Sex (M:F)	17:5 (77%: 23%)
aaCCI (median)	6 (3–9)
cFS (median)	3 (2–4)
Subsite	Oral Cavity—16 (73%) Oropharynx—6 (27%)
Histology	SCC—18 (82%) MC—2 (9%) ACC—2 (9%)
Stage	II or III—6 (27%) IVA—11 (50%) IVB—4 (18%) IVC—1 (5%)
Radiation	Definitive—8 (27%) Adjuvant—16 (73%)
Concurrent systemic therapy	Yes—12 (55%) No—10 (45%)
Systemic therapy agents	CDDP—9 (75%) Nimotuzumab—2 (17%) Pacli + Carbo—1 (8%)

aaCCI—age adjusted Charlson’s Comorbidity Index, cFS—clinical Frailty scale, SCC—Squamous cell carcinoma, MC- Mucoepidermoid carcinoma, ACC—Adenoid cystic carcinoma, CDDP—Cisplatin, Pacli—Paclitaxane, Carbo—Carboplatin

Nine (41%) patients were elderly, aged ≥ 65 years, all of whom had ≥ 1 comorbidity viz., diabetes, hypertension and, or hypothyroidism. Sixteen (73%) patients had OC cancer, while the remaining 27% had OPx malignancies. The details of histology, subsite, indication and concurrent systemic treatment (CST) are also detailed in Table 1. Two patients with OC cancer received definitive radiation due to unwillingness for surgery/inoperable disease and the rest received adjuvant radiation. Twelve (55%) patients received CST.

Dosimetric comparison between HT and PT plans with the dose received by target volumes and OARs is summarized in Table 2.

**Table 2:** Dosimetry and dose reduction with intensity modulated proton therapy (IMPT)

	IMPT	IMRT	Average of % difference
HR PTV D95 (Avg)	97%	98%	NS
HR CTV D98 (Avg)	98%	100%	NS
LR PTV D95 (Avg)	97%	98%	NS
LR CTV D98 (Avg)	99%	100%	NS
Oral mucosa D mean (Avg)	30.42 GyE	38.05 Gy	13.5% reduction
Spared oral mucosa D mean (Avg)	19.17 GyE	32.16 Gy	38.6% reduction
Spinal cord D max (Avg)	19.64 GyE	35.76 Gy	44.4% reduction
Brainstem D max (Avg)	25.86 GyE	42.03 Gy	36.7% reduction
C/L parotid D mean (Avg)	20.56 GyE	25.26 Gy	28.3% reduction

HR PTV—High risk planning target volume, HR CTV—High risk clinical target volume, LR PTV—Low risk planning target volume, LR CTV—Low risk clinical target volume, D95—Dose received by 95% of volume, D98—Dose received by 98% of volume, Avg—Average, Max—Maximum, C/L—Contralateral, NS—Not Significant, Gy—Gray, GyE Gray equivalent

The coverage of the target volumes by 95% and 98% of the prescription dose was comparable in HT and IMPT plans, for HRCTV and LRCTV, respectively (Table 2).

**Fig. 1:**
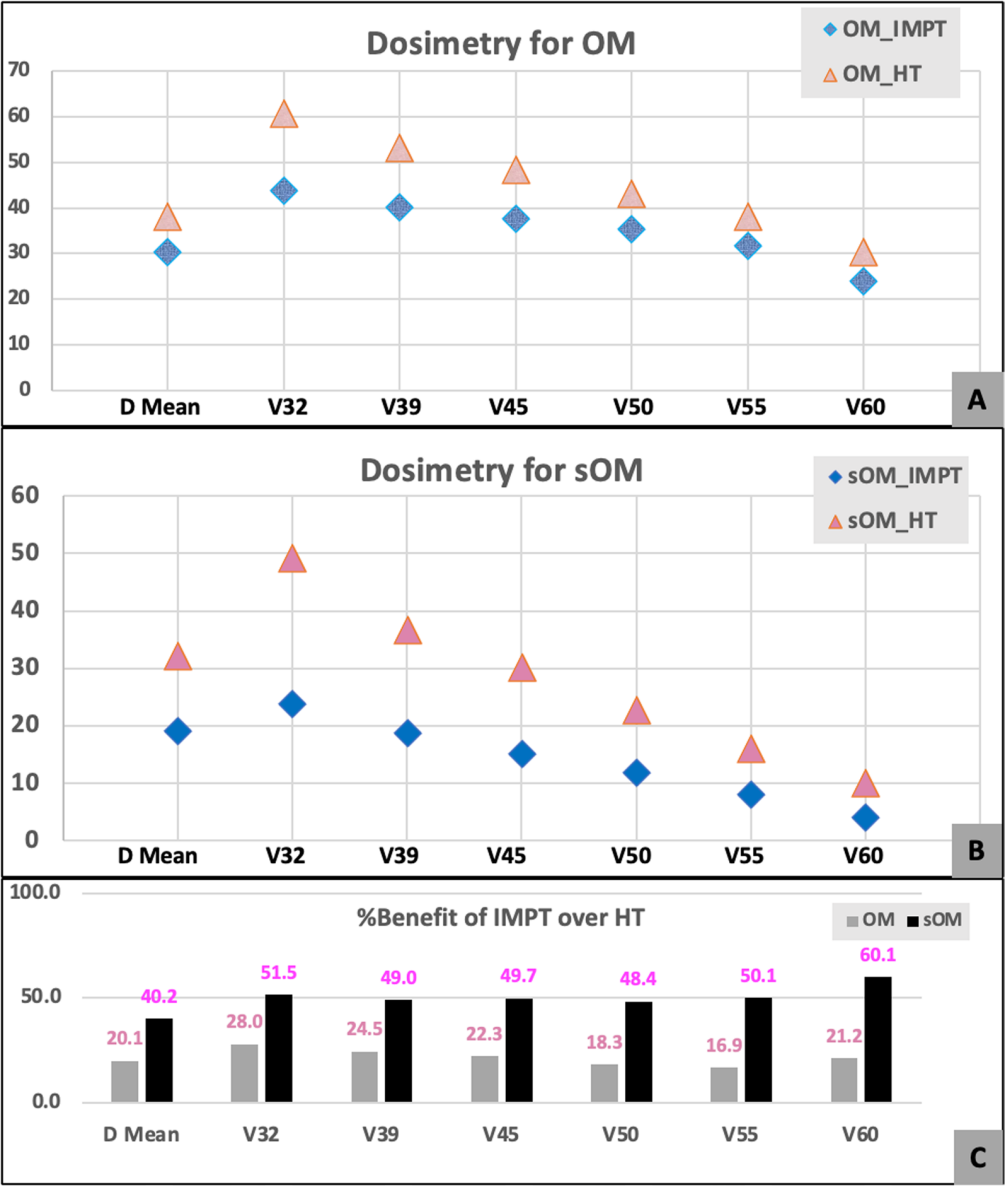
Dosimetric comparison of IMPT and HT. **A**—For Oral mucosa (OM). **B**—For spared oral mucosa (sOM). **C**—Percentage benefit of IMPT over HT for OM and sOM

The average sOM volume was 74% of the total OM volume (range 43 -100). The average mean dose received by the OM was significantly less in IMPT plans (19.17GyE Vs 30.42GyE), while it was further reduced for sOM (19.17GyE with IMPT Vs 32.16 Gy). The volume of OM and sOM receiving 32GyE [V32], 39GyE [V39], 45GyE [V45], 50GyE [V50] and 55GyE [V55] were statistically significantly less in IMPT compared to HT plans [p < 0.05], again better for sOM compared to OM as noted in Fig. 1. The V60 was equivalent for both plans.

The dosimetric advantage of IMPT was maintained for other OARs (Table 2).

**Fig. 2:**
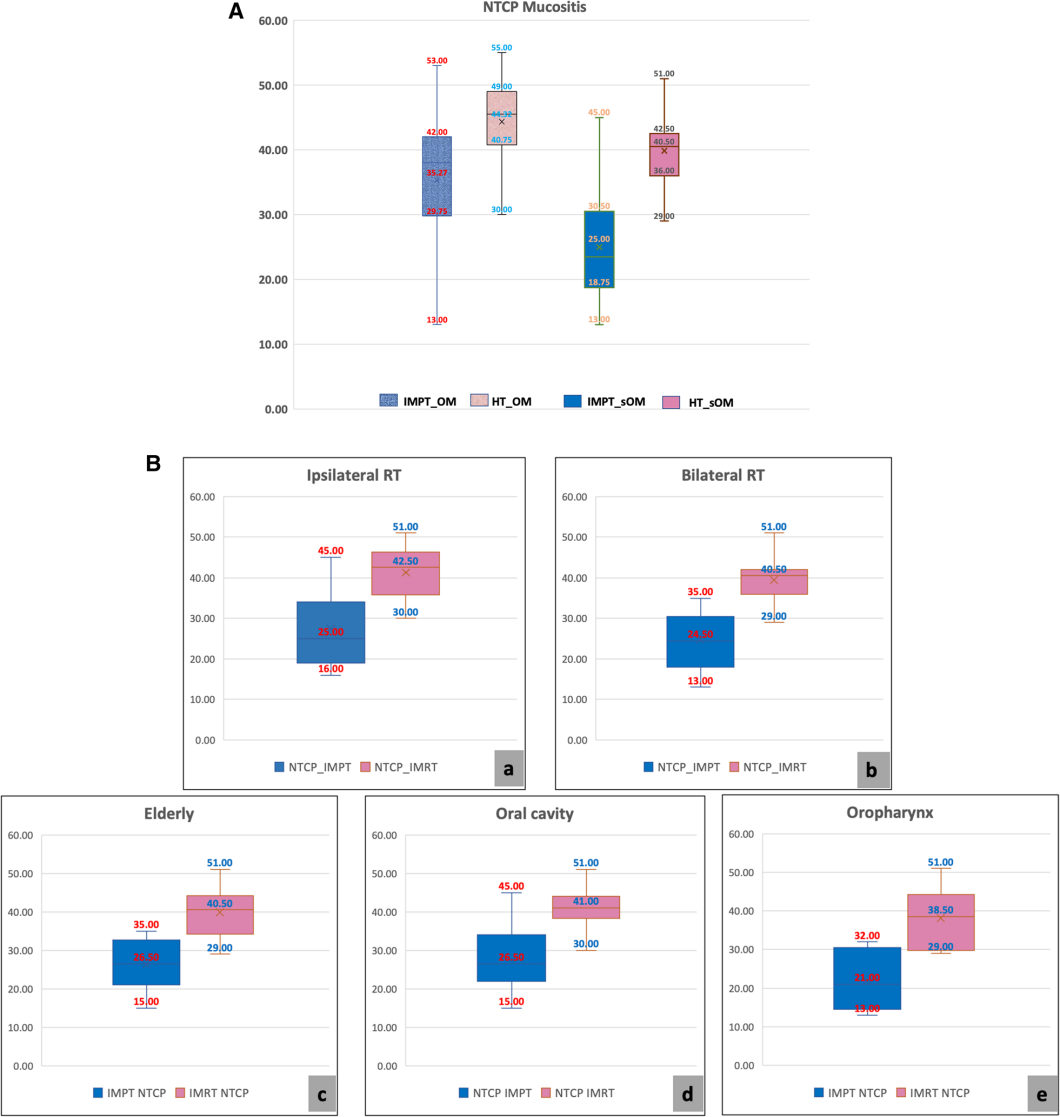
NTCP of mucositis comparison. **A**—NTCP of mucositis for OM, sOM with HT and IMPT, respectively, with the median value for each. **B**—NTCP of mucositis for OM in various subgroups; a—ipsilateral radiation, b—bilateral radiation, c—elderly, d—oral cavity, f—oropharyngeal with  the median value for each

The NTCP for RIOM for both OM and sOM was significantly less with IMPT. With IMPT, NTCP for ≥ grade 3 mucositis based on OM was 38.5 compared to 45.5 with HT, 7% absolute difference, and an average 16.5% relative reduction (Table 3). The reduction in absolute risk in NTCP for RIOM for OM was maintained in subgroup analysis, 7.5% in OC, 8% in OPx and elderly patients, 6.2% in unilateral radiation [RT] and 7% in bilateral RT. The relative benefit ranged from 22.5% to 31.3%, (Fig. 2c) IMPT further reduced NTCP for sOM compared to HT, [24.5 vs 41, 16.6% absolute difference and an average 37% reduction] and also in all subgroups mentioned above (Table 3).

**Table 3:** NTCP of mucositis for oral mucosa and spared oral mucosa with benefit of IMPT

	NTCP for OM		Benefit of IMPT for OM Absolute % (Avg %)	NTCP for sOM		Benefit of IMPT for sOM Absolute % (Avg %)
	IMPT	HT		IMPT	HT	
Overall	38.5	45.5	7.0 (16.3)	24.5	41	16.5 (37)
OC	39.5	47	7.5 (26.5)	27.0	40.8	12.2 (34.5)
OPx	31.0	39.0	8.0 (31.3)	22.0	38.2	16.2 (43.4)
Unilateral RT	36.6	42.8	6.2 (27.7)	27.4	41.3	13.9 (34.8)
Bilateral RT	38.5	45.5	7.0 (24.4)	24.7	39.5	14.8 (38.2)
Elderly	36.5	44.5	8.0 (22.5)	26.3	39.8	13.5 (34.6)

NTCP—Normal tissue complication probability, OM—oral mucosa, sOM—spared oral mucosa, OC—Oral Cavity, OPx—Oropharynx, RT—Radiotherapy, IMPT—Intensity Modulated radiotherapy, HT—Helical Tomotherapy

Acute toxicities are summarized in Table 4. Grade 3 dermatitis and mucositis were noted in 50% and 45.5% of the total population, 11 and 10 patients respectively. Grade 2 mucositis or more was not noted outside the target volume as assessed on clinical examination. Five patients had grade 3 dysphagia, i.e., required feeding-tube insertion; during treatment in 3 and immediately after treatment completion in 2. All patients who required feeding-tube insertion were elderly with ≥ 1 comorbidity.

**Table 4:** Acute toxicities and important parameters

Acute toxicities	Dermatitis	Grade 3—11 / 22 (50%)
	Mucositis (within target)	Grade 3—10 / 22 (45.5%)
	Mucositis (outside target)	Grade 2 or more—None
	Dysphagia	Grade 3—5/ 22 (22.7%)
Median weight loss		2.8 kg
NGT insertion		Yes 6/22, (27.3%) Avg duration of NGT - 8 days (5–16 days)
Unplanned treatment breaks		Yes 8/22, (36.4%) Avg treatment gap - 1.2 days
Hospital admissions during treatment		Yes—5/22, (22.7%) Avg duration of hospital admission - 3.6 days (2–6 days)
Opioid analgesic requirement		Yes—13/ 22 (59.1%)
OTT prolongation		Nil

Avg—Average, NGT—Naso-gastric tube, OTT—Overall treatment time

Five patients required hospital admission during treatment, with an average hospital stay of 3 days. The median weight loss in this cohort was 3.8 kg [1.4 to 9.1 kg]. No patient lost ≥ 10% body weight. Though 8 patients had unplanned treatment gaps of one [6/8] or two [2/8] days, OTT was not prolonged; Treatment acceleration to 6 fractions per week was possible in the latter half of treatment in 7 patients.

At a median follow-up of 15 months, 18 [81.8%] patients of this cohort were alive, 16 [72.7%] were alive without disease, and 2 [9%] were alive with disease. The cause of death was progressive local disease in 1 patient, local and distant progression in 2 and metastatic disease in 1 patient.

## Discussion

We have demonstrated significant sparing of the oral mucosa in IMPT plans compared to HT plans, translating into significantly lower NTCP for RIOM in patients receiving radiation for OC and OPx cancers. We have also assessed control rates and acute toxicity in this group of patients treated with IMPT.

### Oral mucosa delineation and dose–response relationship

The development of RIOM is multifactorial and includes patient factors such as age, sex, comorbidities, immunity, radiosensitivity and treatment factors such as radiation dose, concurrent and, or neoadjuvant systemic therapy [1, 22–24]. RIOM is limited to intermediate and high dose volumes of radiation, usually starting at a cumulative dose of 30 Gy [10]. Given its association with multiple side effects, conformal avoidance of the OM merits the same attention as sparing salivary and dysphagia associated structures. The benefit of sparing of OM has been demonstrated in a randomized trial, by Wang et al. who treated tongue cancer patients with IMRT, and compared sparing the OM outside PTV in the study arm to a mean dose of 32 Gy versus no constraint for this structure in the control arm. They noted significant reduction of grade 2 and 3 mucositis on prescribing a constraint, which resulted in significantly reduced requirement of analgesics and intravenous antibiotics during treatment [8].

Estimation of the probability of oral mucositis on the basis of the dose received by the OM requires uniformity in delineation. The absence of this has been an issue while establishing a pertinent dose-volume relationship. A number of groups have delineated the OC in total, and used it as a surrogate for the OM [20]. The definition of OM proposed by Dean et al. limits this variability by delineating a 3 mm thick oral mucosal surface over the palate, lip, bilateral gingiva [buccal, alveolar, lingual and gingiva proper], tongue and floor of the mouth [FOM] [11]. Compared to the delineation of the OC as an OAR, this mucosal surface volume ensures the inclusion of palatal and buccal mucosal surfaces and the exclusion of tongue and FOM musculature.

Using the guidelines proposed by Dean et al. to delineate OM and then identifying sOM is relevant as different planning interventions and evaluation criteria are required for the OM versus sOM. The only intervention relevant for the OM within the PTV is avoiding hot spots, while the sOM can be prescribed stringent dose constraints. In this context, our average sOM volume, 75% of OM [range 43–93%], allowed the sparing of a substantial part of the oral mucosal surface.

A dose constraint for OM, RIOM being the endpoint, proposed by Narayan et al., was based on a prospective clinical study of 12 HNC patients. The authors noted that a cumulative dose of < 32 Gy and > 39 Gy was associated with minimal acute mucositis and a longer duration of mucositis, respectively [7].

Following this, various authors have established dose–response relationships and predictors for RIOM, such as V30 more than 73%, D21 cc > 10.25 Gy / week and DMean OM [8, 9, 25]. Mazzola et al. have studied the risk of ≥ grade 2 mucositis and identified concurrent chemo-radiotherapy and certain dose parameters pertaining to both the total OM and the spared OM [26]. The relevant parameters pertaining to ≥ grade II and OM out of PTV were V45 > 40%, V50 > 30%, V55 > 20%. An extensive dose–response correlation was also conducted by Bhide et al. [27].

Protons, with their characteristic physical properties, deposit energy at a defined depth i.e., Bragg peak followed by a sharp dose fall off. The dose conformality and convenience of PBS PT allows better sparing of OARs compared to IMRT, and hence encourages its use in HNCs [17, 28–31]. We have applied the same for the HNC cohort [OC and OPx] prevalent in our population.

Our findings, summarized in Tables 2 and 3, showed significant reduction in the dose received by the OM and sOM at all dose points previously identified as significant; DMean, V32, V39, V45, V50, and V55 with PT (Fig. 1; Figs. 5, and 6 in Additional file 1). This reduction ranges from 40 to 60% for the sOM and 16.9% to 28% for the OM as a whole. We have noted this reduction in patients treated for unilateral as well as bilateral targets, as well as for both, OC and OPx cancer patients. Romesser et al., have demonstrated a similar reduction of OC doses, with PT, in a non-randomized comparison of patients receiving unilateral radiotherapy, significant reduction in oral mucositis and dysgeusia [17]. Similarly, the clinical relevance of this dose reduction has been demonstrated for oropharyngeal cancers by Sharma et al., who reported better patient-related outcomes measures [PROMs], pertaining to xerostomia and role function in patients treated with PT for post-op OPx cancers when compared with IMRT, even 12 months after treatment [30].

### NTCP for grade 3 mucositis

NTCP estimation is a tool for predicting treatment-induced toxicities. An ideal NTCP model should be sensitive to non-linear dose–response relationships and multiple interplays between factors; it should be generalisable and should help in predicting various grades of complications at various time points [32].

The NTCP model-based approach was used by Langendijk et al. to identify patients likely to benefit from reduction in NTCP for xerostomia, dysphagia and risk of tube feeding insertion and maximum benefit noted in locally advanced nasopharyngeal and OPx cancers [16]. OC cancers constituted 6% patients in this analysis[16]. Our study illustrates the advantage of proton therapy for cancers prevalent in our population i.e., OC and OPx cancers, and focuses on another clinically relevant endpoint, RIOM.

We have used the NTCP model for oral mucositis proposed by Bhide et al., classified as TRIPOD 4b (TRIPOD criteria—Transparent reporting of multivariate prediction model for individual prognosis or diagnosis), i.e., this model has been validated in an external cohort, though with some caveats, as noted below [34]. This model was created on patients of laryngeal and hypopharyngeal cancers treated in the setting of induction and concurrent chemotherapy [CCT], dose escalation and altered fractionation. It has then been validated by Sharabiani et al., in a cohort treated in a DAHANCA trial for laryngeal and oropharyngeal cancers with standard fractionation and CCT, with or without nimorazole, with acceptable concordance with a Brier score of 0.67 [33]. Subsequently, this has been validated for proton therapy by Blanchard et al. with acceptable concordance, albeit with a 0.17 difference in AUC between the proton cohort and the cross-validated photon cohort, underlining the importance of validation when applying the model across techniques 3dCRT, IMRT and PT [34, 35].

We have calculated NTCP of oral mucositis for the OM and sOM for both IMPT and HT plans. The average absolute difference in NTCP of mucositis [ΔNTCP] for OM was 11.05%, a 20.4% benefit over IMRT, while for ΔNTCP sOM was 14.9%, a 37.3% benefit of IMPT over IMRT (Fig. 2A). The NTCP advantage was maintained for IMPT for both OM and sOM for unilateral and bilateral irradiation, OC and OPx cancers and in elderly patients (Fig. 2B), conclusively demonstrating the advantage of proton therapy.

In our cohort, as summarized in Table 4, 50% and 45.5% patients developed grade 3 dermatitis and mucositis, respectively. We prospectively recorded the location of mucositis within and outside the target based on clinical examination and noted the absence of mucositis outside the target in most patients. On comparison with calculated NTCP results for PT for OM, 38.5%and sOM, 24.5%, we noted a higher incidence of grade 3 oral mucositis (45.5%) entirely within the target volume and none outside. The discordance in calculated NTCP for OM and the actual results can be attributed to use of CST, associated comorbidities, especially diabetes-mellitus and connective tissue disorders, age, immunity, individual radiosensitivity and possible different biological effects of protons; none of these is factored into current NTCP models for mucositis. Blanchard et al. have commented on this discrepancy between calculated NTCP and actual complications, within their group, though maintaining the validity of using NTCP calculation for comparison of plans. However, as noted below, the tolerance of treatment was better than that in historical and contemporary cohorts.

Our tube [NGT]insertion approach was reactive and required in 27.3% patients, exclusively in elderly patients with comorbidities, age being a known risk factor [36]. This compares favorably, at an 11–33% rate of reactive, rather than prophylactic, tube placement rate noted in studies on IMRT [36, 37]. The duration of tube feeding is remarkably short at an average of 8 days [range 5–16]. Beadle et al., reporting on a large SEER-Medicare database have noted a median duration of 100 days, following reactive tube placement.

In a systematic review of 33 randomized trials in HNC, Trotti et al. have noted an average hospital admission duration of 35–42 days; in our cohort it was average 3.6 days [range 2–6], none for mucositis [38]. The median weight loss 2.8 kg [1.5 to 6.8], with no patient losing > 10%, compared to 17% reported noted by Trotti et al. The average unplanned treatment gap was 1.2 days, all patients, including the elderly, completing treatment without prolongation of OTT. At a median follow-up of 12 months, 18/22 [82%] patients of our cohort, comprising 73% patients with Stage IV disease and 41% elderly with comorbidities, were alive.

The limitation of our study is its preliminary nature; validation in a larger, more diverse population, as well as correlation with QOL, will reinforce the benefit of proton therapy in these head-neck subsites. We noted a discrepancy between calculated and actual NTCP for mucositis, although the latter was confined within the target. NTCP models for mucositis require to be more broad-based, incorporating multiple factors noted above.

Long-term efficacy and safety data for the use of PT for HNC is now available [29]. Various health systems have evolved differing strategies for allocating patients for proton therapy. The model-based selection used by the Dutch group has been referred to above [15]. Accrual to a multi-institutional randomized trial has recently been completed, and results are awaited. Our allocation for this cohort has been based on dosimetric superiority. HNC patients undergoing radiotherapy are being enrolled on the prospective HenckQOL registry for assessment of outcomes and QOL to study this further.

Our results demonstrate the importance of radiation techniques in improving the tolerance of radiation and the benefit of modern proton therapy in OC and OPx patients, the former especially relevant in South Asia, and not addressed so far in reference to proton therapy. Improved tolerance of treatment, even with > 40% population in this cohort being elderly, reinforces the impact of sparing the oral mucosa by proton therapy.

## Conclusion

In conclusion, in all age groups, NTCP for mucositis is significantly less with IMPT for OC and OPx cancers. The acute toxicity profile of our patients encourages the use of this modality for improving the therapeutic ratio of HNC treatment.

## Supplementary Information

**Additional file 1.** Supplementary File.

## Data Availability

Data will be made available upon request. Data sharing: The data have not been deposited in a repository at present but are available for scrutiny by reviewers, if required.

## References

[1] Bowen J, Al-Dasooqi N, Bossi P, Wardill H, Van Sebille Y, Al-Azri A, Bateman E, Correa ME, Raber-Durlacher J, Kandwal A, Mayo B, Nair RG, Stringer A, Ten Bohmer K, Thorpe D, Lalla RV, Sonis S, Cheng K, Elad S (2019). Mucositis Study Group of the Multinational Association of Supportive Care in Cancer/International Society of Oral Oncology (MASCC/ISOO). The pathogenesis of mucositis: updated perspectives and emerging targets. Support Care Cancer.

[2] Pulito C, Cristaudo A, Porta C, Zapperi S, Blandino G, Morrone A, Strano S (2020). Oral mucositis: the hidden side of cancer therapy. J Exp Clin Cancer Res.

[3] Bar AdV, Weinstein G, Dutta PR, Dosoretz A, Chalian A, Both S, Quon H (2010). Gabapentin for the treatment of pain syndrome related to radiation-induced mucositis in patients with head and neck cancer treated with concurrent chemoradiotherapy. Cancer.

[4] Muzumder S, Nirmala S, Avinash HU, Sebastian MJ, Kainthaje PB (2018). Analgesic and opioid use in pain associated with head-and-neck radiation therapy. Indian J Palliat Care..

[5] Muzumder S, Srikantia N (2020). Toxicity syndrome and early competing deaths in head-and-neck cancer undergoing radiation therapy: observation and hypothesis. Med Hypotheses.

[6] Maria OM, Eliopoulos N, Muanza T (2017). Radiation-induced oral mucositis. Front Oncol.

[7] Narayan S, Lehmann J, Coleman MA, Vaughan A, Yang CC, Enepekides D, Farwell G, Purdy JA, Laredo G, Nolan K, Pearson FS, Vijayakumar S (2008). Prospective evaluation to establish a dose response for clinical oral mucositis in patients undergoing head-and-neck conformal radiotherapy. Int J Radiat Oncol Biol Phys.

[8] Wang ZH, Zhang SZ, Zhang ZY, Zhang CP, Hu HS, Tu WY, Kirwan J, Mendenhall WM (2012). Protecting the oral mucosa in patients with oral tongue squamous cell carcinoma treated postoperatively with intensity-modulated radiotherapy: a randomized study. Laryngoscope.

[9] Otter S, Schick U, Gulliford S, Lal P, Franceschini D, Newbold K, Nutting C, Harrington K, Bhide S (2015). Evaluation of the risk of grade 3 oral and pharyngeal dysphagia using atlas-based method and multivariate analyses of individual patient dose distributions. Int J Radiat Oncol Biol Phys.

[10] Musha A, Shimada H, Shirai K, Saitoh J, Yokoo S, Chikamatsu K, Ohno T, Nakano T (2015). Prediction of acute radiation mucositis using an oral mucosal dose surface model in carbon ion radiotherapy for head and neck tumors. PLoS ONE.

[11] Dean JA, Welsh LC, Gulliford SL, Harrington KJ, Nutting CM (2015). A novel method for delineation of oral mucosa for radiotherapy dose-response studies. Radiother Oncol..

[12] Wang X, Eisbruch A (2016). IMRT for head and neck cancer: reducing xerostomia and dysphagia. J Radiat Res..

[13] López Alfonso JC, Parsai S, Joshi N, Godley A, Shah C, Koyfman SA, Caudell JJ, Fuller CD, Enderling H, Scott JG (2018). Temporally feathered intensity-modulated radiation therapy: a planning technique to reduce normal tissue toxicity. Med Phys.

[14] Cozzi L, Fogliata A, Lomax A, Bolsi A (2001). A treatment planning comparison of 3D conformal therapy, intensity modulated photon therapy and proton therapy for treatment of advanced head and neck tumours. Radiother Oncol.

[15] Langendijk JA, Lambin P, De Ruysscher D, Widder J, Bos M, Verheij M (2013). Selection of patients for radiotherapy with protons aiming at reduction of side effects: the model-based approach. Radiother Oncol.

[16] Tambas M, Steenbakkers RJHM, van der Laan HP, Wolters AM, Kierkels RGJ, Scandurra D, Korevaar EW, Oldehinkel E, van Zon-Meijer TWH, Both S, van den Hoek JGM, Langendijk JA (2020). First experience with model-based selection of head and neck cancer patients for proton therapy. Radiother Oncol.

[17] Romesser PB, Cahlon O, Scher E, Zhou Y, Berry SL, Rybkin A, Sine KM, Tang S, Sherman EJ, Wong R, Lee NY (2016). Proton beam radiation therapy results in significantly reduced toxicity compared with intensity-modulated radiation therapy for head and neck tumors that require ipsilateral radiation. Radiother Oncol..

[18] Nangia S, Chufal KS, Arivazhagan V, Srinivas P, Tyagi A, Ghosh D (2006). Compensator-based intensity-modulated radiotherapy in head and neck cancer: our experience in achieving dosimetric parameters and their clinical correlation. Clin Oncol (R Coll Radiol).

[19] Nangia S, Chufal KS, Tyagi A, Bhatnagar A, Mishra M, Ghosh D (2010). Selective nodal irradiation for head and neck cancer using intensity-modulated radiotherapy: application of RTOG consensus guidelines in routine clinical practice. Int J Radiat Oncol Biol Phys.

[20] Brouwer CL, Steenbakkers RJ, Bourhis J, Budach W, Grau C, Grégoire V, van Herk M, Lee A, Maingon P, Nutting C, O'Sullivan B, Porceddu SV, Rosenthal DI, Sijtsema NM, Langendijk JA (2015). CT-based delineation of organs at risk in the head and neck region: DAHANCA, EORTC, GORTEC, HKNPCSG, NCIC CTG, NCRI, NRG Oncology and TROG consensus guidelines. Radiother Oncol.

[21] Dean JA, Wong KH, Gay H, Welsh LC, Jones AB, Schick U, Oh JH, Apte A, Newbold KL, Bhide SA, Harrington KJ, Deasy JO, Nutting CM, Gulliford SL (2016). Functional data analysis applied to modeling of severe acute mucositis and dysphagia resulting from head and neck radiation therapy. Int J Radiat Oncol Biol Phys..

[22] Sonis ST (2009). Mucositis: the impact, biology and therapeutic opportunities of oral mucositis. Oral Oncol.

[23] Werbrouck J, De Ruyck K, Duprez F, Veldeman L, Claes K, Van Eijkeren M, Boterberg T, Willems P, Vral A, De Neve W, Thierens H (2009). Acute normal tissue reactions in head-and-neck cancer patients treated with IMRT: influence of dose and association with genetic polymorphisms in DNA DSB repair genes. Int J Radiat Oncol Biol Phys.

[24] Citrin DE, Mitchell JB (2017). Mechanisms of normal tissue injury from irradiation. Semin Radiat Oncol.

[25] Li K, Yang L, Hu QY, Chen XZ, Chen M, Chen Y (2017). Oral mucosa dose parameters predicting grade ≥3 acute toxicity in locally advanced nasopharyngeal carcinoma patients treated with concurrent intensity-modulated radiation therapy and chemotherapy: an independent validation study comparing oral cavity versus mucosal surface contouring techniques. Transl Oncol..

[26] Mazzola R, Ricchetti F, Fersino S, Fiorentino A, GiajLevra N, Di Paola G, Ruggieri R, Alongi F (2016). Predictors of mucositis in oropharyngeal and oral cavity cancer in patients treated with volumetric modulated radiation treatment: a dose-volume analysis. Head Neck.

[27] Bhide SA, Gulliford S, Schick U, Miah A, Zaidi S, Newbold K, Nutting CM, Harrington KJ (2012). Dose-response analysis of acute oral mucositis and pharyngeal dysphagia in patients receiving induction chemotherapy followed by concomitant chemo-IMRT for head and neck cancer. Radiother Oncol.

[28] McKeever MR, Sio TT, Gunn GB, Holliday EB, Blanchard P, Kies MS, Weber RS, Frank SJ (2016). Reduced acute toxicity and improved efficacy from intensity-modulated proton therapy (IMPT) for the management of head and neck cancer. Chin Clin Oncol.

[29] Gunn GB, Garden AS, Ye R, Ausat N, Dahlstrom KR, Morrison WH, Fuller CD, Phan J, Reddy JP, Shah SJ, Mayo LL, Chun SG, Chronowski GM, Moreno AC, Myers JN, Hanna EY, Esmaeli B, Gillison ML, Ferrarotto R, Hutcheson KA, Chambers MS, Ginsberg LE, El-Naggar AK, Rosenthal DI, Zhu XR, Frank SJ (2021). Proton therapy for head and neck cancer: a 12-year. Single-institution experience. Int J Part Ther.

[30] Sharma S, Zhou O, Thompson R, Gabriel P, Chalian A, Rassekh C, Weinstein GS, O'Malley BW, Aggarwal C, Bauml J, Cohen RB, Lukens JN, Swisher-McClure S, Ghiam AF, Ahn PH, Lin A (2018). Quality of life of postoperative photon versus proton radiation therapy for oropharynx cancer. Int J Part Ther.

[31] Moreno AC, Frank SJ, Garden AS, Rosenthal DI, Fuller CD, Gunn GB, Reddy JP, Morrison WH, Williamson TD, Holliday EB, Phan J, Blanchard P (2019). Intensity modulated proton therapy (IMPT)—the future of IMRT for head and neck cancer. Oral Oncol..

[32] Van den Bosch L, Schuit E, van der Laan HP, Reitsma JB, Moons KGM, Steenbakkers RJHM, Hoebers FJP, Langendijk JA, van der Schaaf A (2020). Key challenges in normal tissue complication probability model development and validation: towards a comprehensive strategy. Radiother Oncol.

[33] Sharabiani M, Clementel E, Andratschke N, Hurkmans C (2020). Generalizability assessment of head and neck cancer NTCP models based on the TRIPOD criteria. Radiother Oncol.

[34] Sharabiani M, Clementel E, Andratschke N, Collette L, Fortpied C, Grégoire V, Overgaard J, Willmann J, Hurkmans C (2021). Independent external validation using the EORTC HNCG-ROG 1219 DAHANCA trial data of NTCP models for acute oral mucositis. Radiother Oncol.

[35] Blanchard P, Wong AJ, Gunn GB, Garden AS, Mohamed ASR, Rosenthal DI, Crutison J, Wu R, Zhang X, Zhu XR, Mohan R, Amin MV, Fuller CD, Frank SJ (2016). Toward a model-based patient selection strategy for proton therapy: External validation of photon-derived normal tissue complication probability models in a head and neck proton therapy cohort. Radiother Oncol.

[36] Sachdev S, Refaat T, Bacchus ID, Sathiaseelan V, Mittal BB (2015). Age most significant predictor of requiring enteral feeding in head-and-neck cancer patients. Radiat Oncol.

[37] Beadle BM, Liao KP, Giordano SH, Garden AS, Hutcheson KA, Lai SY, Guadagnolo BA (2017). Reduced feeding tube duration with intensity-modulated radiation therapy for head and neck cancer: a surveillance, epidemiology, and end results-medicare analysis. Cancer.

[38] Trotti A, Bellm LA, Epstein JB, Frame D, Fuchs HJ, Gwede CK, Komaroff E, Nalysnyk L, Zilberberg MD (2003). Mucositis incidence, severity and associated outcomes in patients with head and neck cancer receiving radiotherapy with or without chemotherapy: a systematic literature review. Radiother Oncol.

