# Supplementary material for: Proton therapy and oral mucositis in oral & oropharyngeal cancers: outcomes, dosimetric and NTCP benefit
Source: Radiat Oncol. 2023 Jul 19;18:121. doi: 10.1186/s13014-023-02317-1 (PMC10357709; doi:10.1186/s13014-023-02317-1)
Supplement: Supplementary file 1 — Additional file 1. Supplementary File. [file 13014_2023_2317_MOESM1_ESM.docx]

Supplementary File.

**Proton therapy planning and target delineation:**

The planning process was initiated after Multi-disciplinary Tumour Board review. Patients were simulated as per institutional protocol, after informed consent and a pre-planning audit. All patients underwent pre-radiotherapy dental assessment and replacement of metallic dental implants with acrylic implants, if possible. Details of implant material (size, type, density) were obtained in patients for whom dental implant removal or replacement was not feasible. Similar details were also sought for reconstruction materials viz., titanium plates, for patients with bucco-alveolar cancers.

**Mouth-bite**

A custom mouth-bite was fabricated with thermoplastic pellets (Adapt-IT, Qfix, Avondale, USA). We used these customized mouth-bites to separate and immobilize the tongue from the target for unilateral irradiation or separate palate and buccal mucosa from central targets. Notwithstanding the sharp dose fall-off in proton therapy, these help to minimize dose and end-ranging on the mucosa distal to the target.

**Mould, Neck rest and CT acquisition**

A customized neck rest (Mouldcare Cushion, Qfix, Avondale, USA) and thermoplastic mask (Fibreplast, Qfix, Avondale, USA) were used for immobilisation. Computed tomography (CT) simulation was performed with 2 mm slice thickness axial images using Canon Aquilion LB CT scanner ( Canon Medical Systems, Singapore). Another CT scan with radio-opaque wires marking the scar, and oral contrast to delineate mouth bite was acquired. An MVCT scan was acquired in planning position on Radixact (V 2.0.1.1, Accuray Inc., Sunnyvale, USA ) machine for better delineation of dental implants, if any.

**Target delineation**

Volumetric images of planning MRI or any pre-op imaging, or both, were registered with planning CT images to aid target and OAR delineation.

The primary gross tumour volume (GTVp) and nodal volume (GTVn) were delineated in all patients undergoing definitive proton therapy. Both, the primary and nodal preoperative GTVs, were delineated in postop patients.

In patients undergoing definitive radiation, the GTV was expanded 10-15 mm and modified for anatomical barriers to generate the primary high risk clinical target volume (HRCTV P). The nodal levels with gross nodes were included in HRCTV (HRCTV N); elective nodal volume was delineated as previously published and designated low risk CTV (LRCTV) (20)(~~Author’s publication-Blinded reference)~~. HRCTV was prescribed a dose of 70GyE (Gray equivalent) and LRCTV 56GyE in 35 fractions. In patients undergoing post-operative radiation the operative bed and flap were included in the high risk CTV and the dose prescribed was 60GyE to HRCTV. The LRCTV was prescribed 54GyE. The simultaneous integrated boost (SIB) technique was used. Post-operative patients with high risk features, i.e., extra nodal extension, close or positive margins, and / or peri neural invasion, received 4-6GyE as sequential boost.

**Proton planning**

Robustly optimized MFO-IMPT plans was created for each patient using four oblique fields, on a RayStation (Version 9.0, RaySearch Laboratories AB, Stockholm, Sweden) treatment planning system (TPS), while patients requiring ipsilateral neck irradiation, were treated with 3 field plans. RayStation TPS has been clinically commissioned for Proteus-Plus (IBA, Louvain-La-Neuve, Belgium) proton delivery system equipped with dedicated pencil beam scanning nozzle, capable of delivering cyclotron produced proton energies from 70.18 to 226.2 MeV. RayStation Monte-Carlo (MC) algorithm was used for optimization of spot position, spot weight, energy layers as well as dose computation. Robust optimization was performed incorporating 3 mm set-up error in all translational axes and a range uncertainty of 3.5% using MiniMax algorithm. A range shifter having water equivalent thickness (WET) of 7.5 g/cm2 was used wherever applicable. Additional attention was given to the region adjacent to dental metal artefacts, taking into account the material used. Treatment plans were evaluated for target coverage & OARs dose, using standard dose volume indices derived from dose-volume histograms (DVH), including worst case scenarios, using robust evaluation as detailed above.

**Tomotherapy planning**

A Helical Tomotherapy (HT) treatment plan was generated for each patient in Precision (V 2.0.1.1, Accuray Inc., Sunnyvale, USA) TPS using 6 MV FFF photon beam with field widths of 1 cm or 2.5 cm and variable pitch and modulation factor.

**Pre Treatment assessments**

All patients underwent Speech and Swallowing Assessment, Nutrition Assessment, nutritional assessment using PGSGA, and baseline quality of life assessment using EORTC questionnaire (C30 and HN35), prior to treatment implementation. Most patients were referred to Clinical Psycho-Therapist for relaxation exercises, as a standard institutional protocol.

The departmental protocol for feeding tube insertion on significant (>10%) weight loss or severe odynophagia (Grade 3 or more), was followed.

**Plan Implementation**

Finalized IMPT plans were implemented after pre-treatment patient specific quality assurance using Proteus Plus under daily CBCT based image guidance. In addition, intra-treatment change in patient motion was monitored using Align RT (Vision RT, Version 5.1.2, London, United Kingdom) based surface guidance. Modest acceleration was practiced, with 6 fractions being delivered per week in the latter two to three weeks of treatment, subject to acute toxicities and overall tolerance.

Quality assurance CT (QACT) scans and assessment of quality of proton therapy plans was conducted at regular intervals. During radiation treatment, all patients were reviewed at least once weekly for acute toxicity assessment.

**Data collection and analysis**

Dosimetric data for all patients collected and compiled using SPSS Software version 21, which was used for statistical analysis also. Dosimetric variables for oral mucosa (OM) and spared oral mucosa (sOM) namely D_Mean, V32, V39, V45, V50, V55, V60 for IMPT and HT plans were compared using paired t test, two tailed significance (p value) was calculated. (Fig 4)

Fig 3


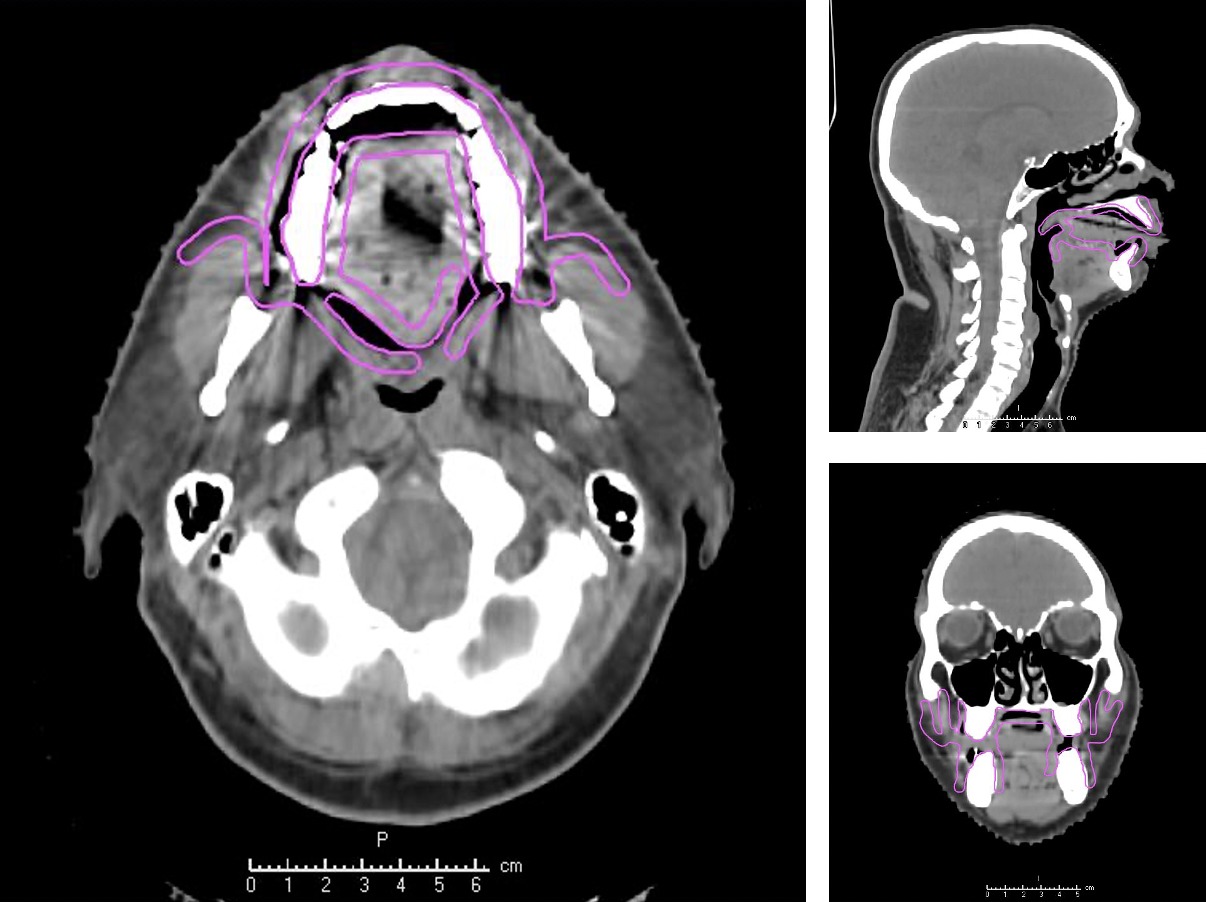


Fig 4


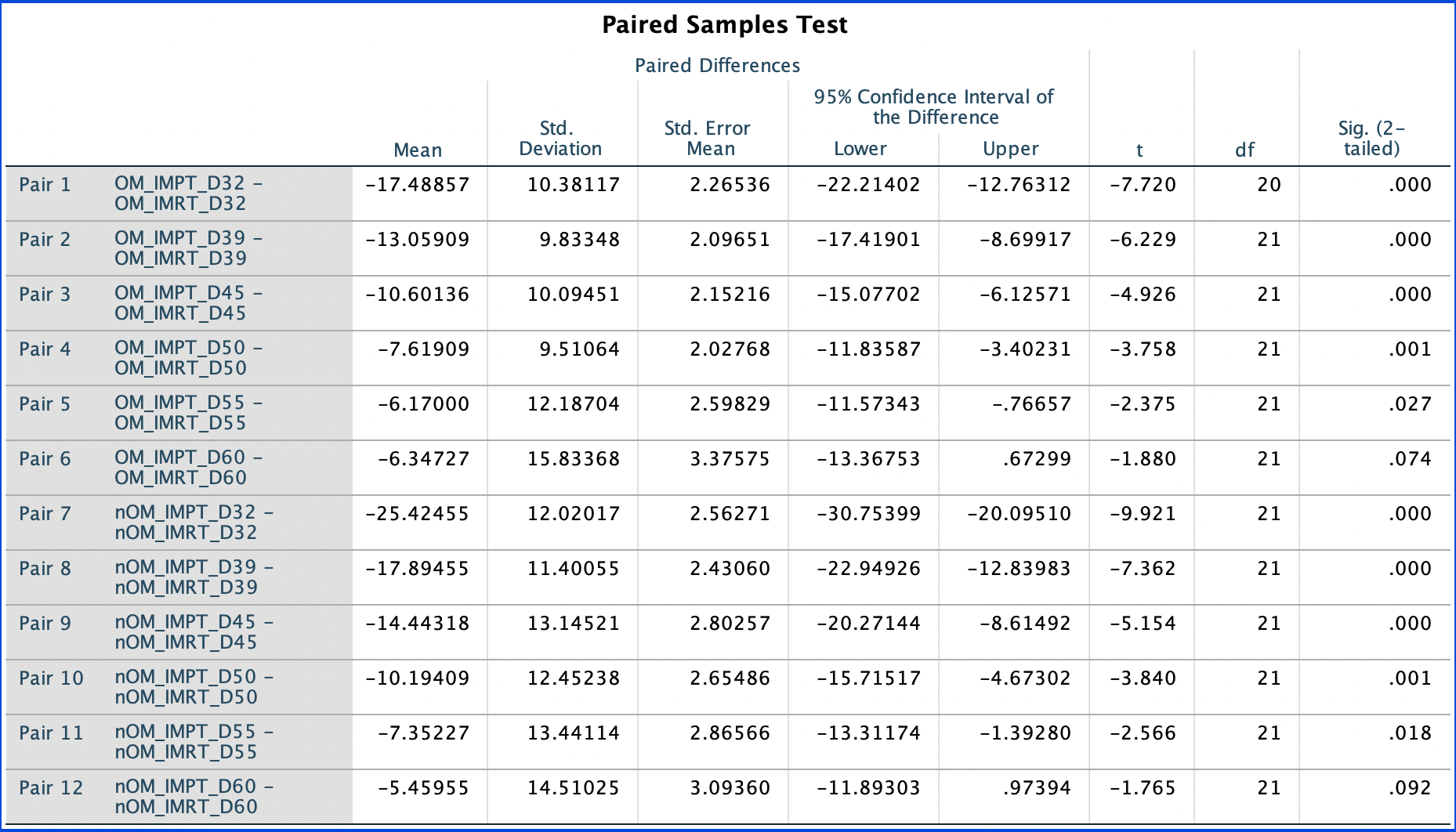


Fig 5


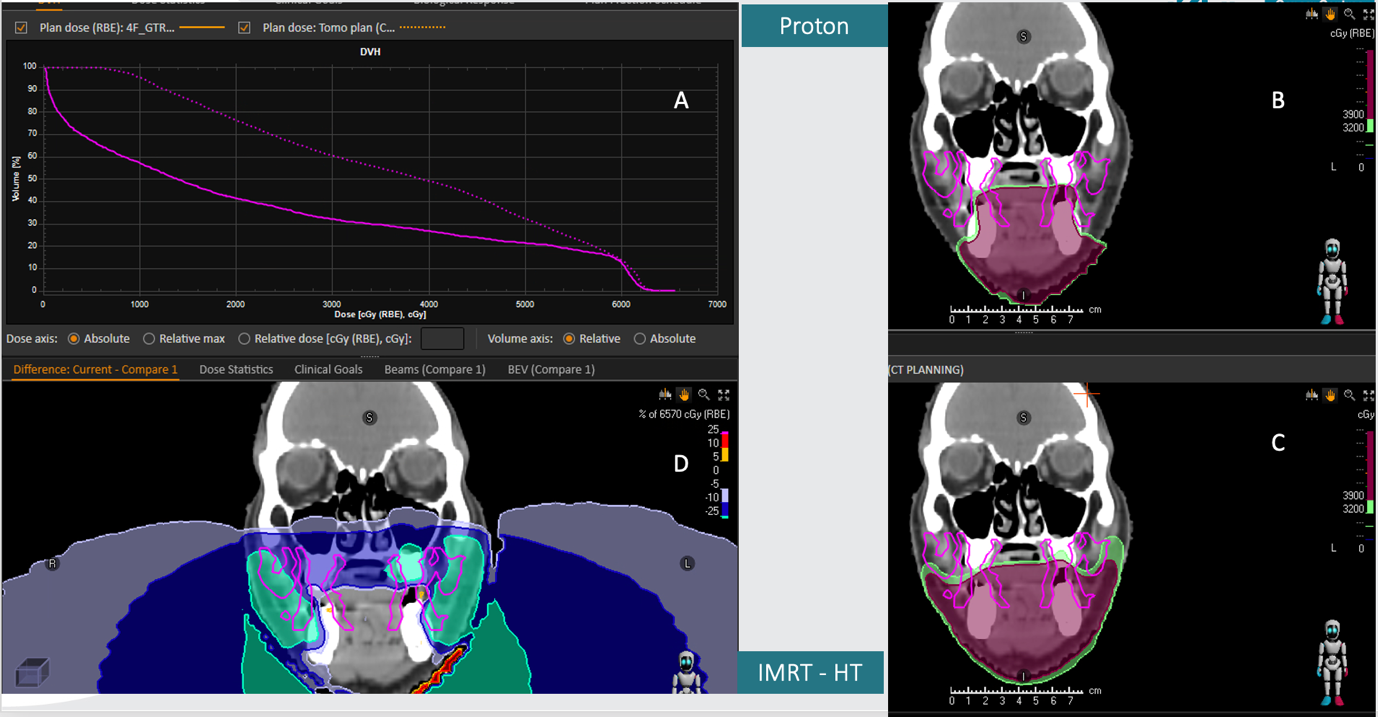


Fig 6


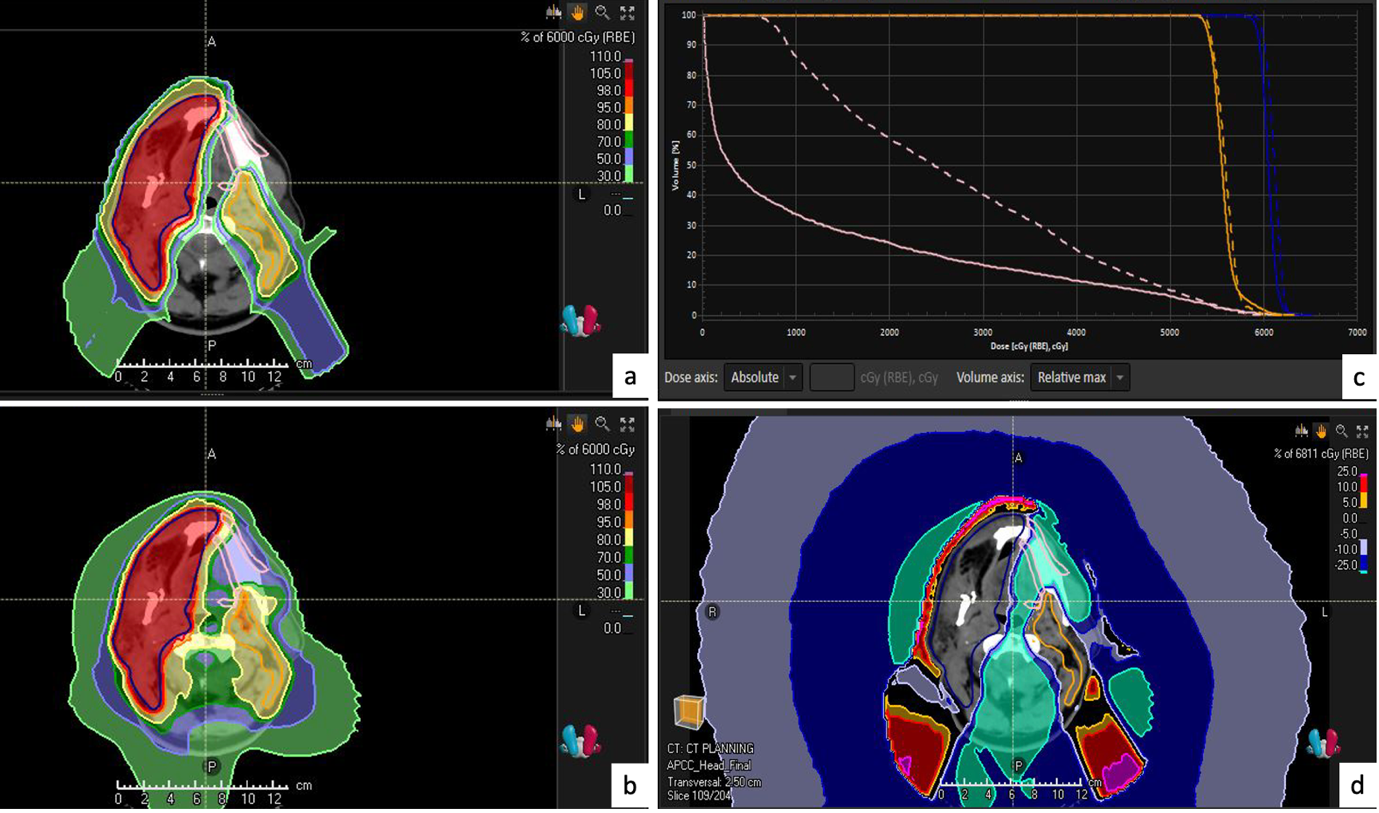


Legends

Fig 3: Delineation of buccal mucosa, as proposed by Dean et al (9)

Fig 4: Paired t-test, demonstrating statistically significant differences in oral mucosa ( OM) and spared oral mucosa( sOM) IMRT and HT plans volumes receiving 32Gy, 39Gy, 45Gy, 50Gy and 55 Gy. The difference if not statistically different for volumes receiving 60Gy.

Fig 5: (A) DVH difference for oral mucosa, IMPT - solid line, Helical Tomotherapy ( HT) - dotted line. Dose colourwash representing 39 Gy ( red) and 32 Gy ( green) sparing oral mucosa, magenta contour, with IMPT ( B), HT (C). (D) Comparison between IMPT and HT

Fig 6: Dose colour wash (dose distribution) comparison of IMPT and HT plans

A – IMPT Plan

B – HT Plan

C – DVH showing the difference in D Mean for OM

D – Dose differences between IMPT and HT plans
